# A new species of sucking louse *Hoplopleura villosissima* n. sp. (Psocodea: Phthiraptera: Hoplopleuridae) and a new host record of the spiny rat louse *Polyplax spinulosa* Burmeister, 1839 (Psocodea: Phthiraptera: Polyplacidae) from the long-haired rat *Rattus villosissimus* Waite (Rodentia: Muridae) in Australia

**DOI:** 10.1186/s13071-018-3037-8

**Published:** 2018-08-23

**Authors:** Wei Wang, Haylee J. Weaver, Fan Song, Lance A. Durden, Renfu Shao

**Affiliations:** 1 GeneCology Research Centre, Centre for Animal Health Innovation, School of Science and Engineering, Faculty of Science, Health, Education and Engineering, University of the Sunshine Coast, Maroochydore, Queensland 4556 Australia; 2 Australian Biological Resources Study, Department of the Environment and Energy, GPO Box 787, Canberra, ACT 2601 Australia; 3 Department of Entomology, China Agricultural University, Beijing, 100193 China; 4 Department of Biology, Georgia Southern University, Statesboro, Georgia 30458 USA

**Keywords:** Phthiraptera, Psocodea, Hoplopleuridae, Polyplacidae, *Hoplopleura villosissima*, *Polyplax spinulosa*, Muridae, *Rattus villosissimus*, *Rattus rattus*

## Abstract

**Background:**

The sucking louse fauna of endemic Australian rodents has been under-studied for decades. Sixty-five species of native rodents have been recorded in Australia. However, only 11 species of lice have been reported from 11 species of endemic Australian rodents.

**Results:**

We describe a new species of sucking louse, *Hoplopleura villosissima* Wang (Psocodea: Phthiraptera: Hoplopleuridae), and report a new host record of the spiny rat louse, *Polyplax spinulosa* Burmeister, 1839 (Psocodea: Phthiraptera: Polyplacidae), from the long-haired rat, *Rattus villosissimus* Waite (Rodentia: Muridae), which is endemic to Australia.

**Conclusions:**

This study is the first record of sucking louse from *R. villosissimus* and the first record of a species of *Polyplax* Enderlein, 1904 from an endemic Australian rodent. This study brings the total number of sucking louse species in endemic Australian rodents from 11 to 13. Previously, only the introduced brown rat, *Rattus norvegicus* Berkenhout and the black rat, *Rattus rattus* Linnaeus were recorded as the hosts of *P. spinulosa* in Australia. Because *R. villosissimus* overlaps with *R. rattus* in distribution but not with *R. norvegicus*, we propose that *P. spinulosa* transferred to *R. villosissimus* from *R. rattus*.

## Background

Sucking lice (Psocodea: Phthiraptera: Anoplura) are obligate, permanent external parasites of eutherian mammals [1]. A number of sucking louse species are known vectors and transmit pathogenic microorganisms in humans, livestock, and wild animals [2]. More than 540 species of sucking lice from 840 species of eutherian mammals have been described [3]. Rodents are the most common hosts of sucking lice. Globally, 40% of rodent species are known to be hosts to sucking lice, and 67% of described sucking louse species parasitize rodents [2, 4]. The two most species-rich genera, *Hoplopleura* Enderlein, 1904 and *Polyplax* Enderlein, 1904, have 136 and 79 species, respectively, almost all parasitizing rodents [3].

Currently biogeographically isolated from the rest of the world, the Australian continent has a distinct endemic fauna of rodents (Muridae) [5, 6]. Sixty-five species of native rodents (including extinct species) have been recorded in Australia. However, only 11 species of lice, all belonging to the family Hoplopleuridae Ewing, 1929, have been recorded from 11 species of native Australian rodents; with seven species recorded before 1972 [7–9] and four species since 2008 [10, 11]. All of the 11 species of sucking lice recorded from Australian rodents are in the genus *Hoplopleura*. No *Polyplax* (family Polyplacidae Fahrenholz, 1912) species have previously been recorded from endemic Australian rodents. The sucking louse fauna of endemic Australian rodents is under-studied, and more species of sucking lice remain to be described [12].

During this study, we collected lice from *Rattus villosissimus* Waite (Rodentia: Muridae), an endemic species to Australia, and *Rattus rattus* Linnaeus (Rodentia: Muridae), an introduced species to Australia after European settlement. We describe a new louse species, *Hoplopleura villosissima*, and provide a new host record of the spiny rat louse, *Polyplax spinulosa* Burmeister, 1839 from *R. villosissimus*. We also propose that *P. spinulosa* transferred from *R. rattus* to *R. villosissimus*.

## Methods

Specimens of lice were collected from voucher specimens of *R. villosissimus* stored in ethanol and accessioned into the Queensland Museum, Brisbane, Australia, and from *R. rattus* collections stored in the Melbourne Museum, Australia. Lice were collected from host pelage using a head louse comb following a modified “cocktail shaking” technique [11]. In brief, rodent specimens were placed on a tray, one at a time, and combed gently and thoroughly. Each rodent was then transferred into a small jar which was filled with 70% ethanol. The jar was sealed with a cap and shaken gently for ~1 min to dislodge lice from the host. After shaking the jar, rodents were removed and the ethanol solution was filtered through a fine mesh. Filtered extracts were examined for lice under a dissecting microscope (Nikon SMZ 800N); lice found were transferred to labelled vials and stored in 100% ethanol at -20 °C.

For morphological examination, lice were mounted on slides using a protocol detailed by Palma [13]. Intact specimens with well-extended legs and antennae and minimal gut contents were selected for mounting. These specimens were immersed in a 20% potassium hydroxide (KOH) solution for 24 to 48 h at room temperature; the ventral surface of abdomen was punctured with a fine micropin and then gently squeezed to expel digested tissues. Specimens were then transferred to distilled water and cleaned for a further 30 min to expel any remaining gut contents. The water was then replaced with 10% acetic acid solution for 1 h. The specimens were stained with 1% acid fuchsin for 2–4 h, and gradually dehydrated in 40%, 70% and then absolute ethanol, each for 30 min. After dehydration, specimens were immersed in pure clove oil for 24 h. The specimens were then mounted on slides, ventral surface up, with a small amount of Canada balsam (kept in xylene). Mounted slides were dried in an oven at 40–45 °C for 2–3 weeks. The morphology of lice was examined using a Photomicroscope (Nikon ECLIPSE T*s* 2). All measurements are in micrometres (range followed by the mean). Descriptive format and abbreviations follow Kim et al. [14], with full names of setae spelled out in full at first mention. Host taxonomy follows Wilson & Reeder [15].

## Results

**Family Hoplopleuridae Ewing, 1929**

**Genus** ***Hoplopleura*** **Enderlein, 1904**

*Hoplopleura villosissima* Wang n. sp.

***Type-host*:** *Rattus villosissimus* (Waite, 1898) (Rodentia: Muridae), long-haired rat.

***Type locality*:** Sandringham (23°56'S, 138°47'E), Queensland, Australia.

***Type-material*:** Holotype male and allotype female ex *Rattus villosissimus* (Queensland Museum QM JM4825, 29.vii.1984, unknown collector): holotype, ♂ (QM T244609), Allotype, ♀ (QM T244610), same data as for the holotype. Paratypes: 1 ♂ (QM T244611) and 2♀ (QM T244612, T244613), same data as for the holotype.

***Additional material examined*:** 2 ♂ and 2♀ ex *Rattus villosissimus* (QM JM4823) and 2♂ and 2 ♀ ex *Rattus villosissimus* (QM JM4824), same location as holotype and 2♂ and 1 ♀ ex *Rattus villosissimus* (QM JM10742), Diamantina Lakes, Queensland, Australia (23°40'S, 141°5'E), 10.viii.1981, unknown collector.

***ZooBank registration*:** To comply with the regulations set out in article 8.5 of the amended 2012 version of the *International Code of Zoological Nomenclature* (ICZN) [16], details of the new species have been submitted to ZooBank. The Life Science Identifier (LSID) of the article is urn:lsid: zoobank.org:pub:3233A8E1-0F17-4395-8517-E48D07D5D1F7. The LSID for the new name *Hoplopleura villosissima* is urn:lsid: zoobank.org:act:D621C005-5E21-4A28-A8A3-D671439D84DB [16].

***Etymology*:** The species epithet is a noun in apposition referring to the specific name of the host species, *Rattus villosissimus*.

### Description

**Fig. 1:**
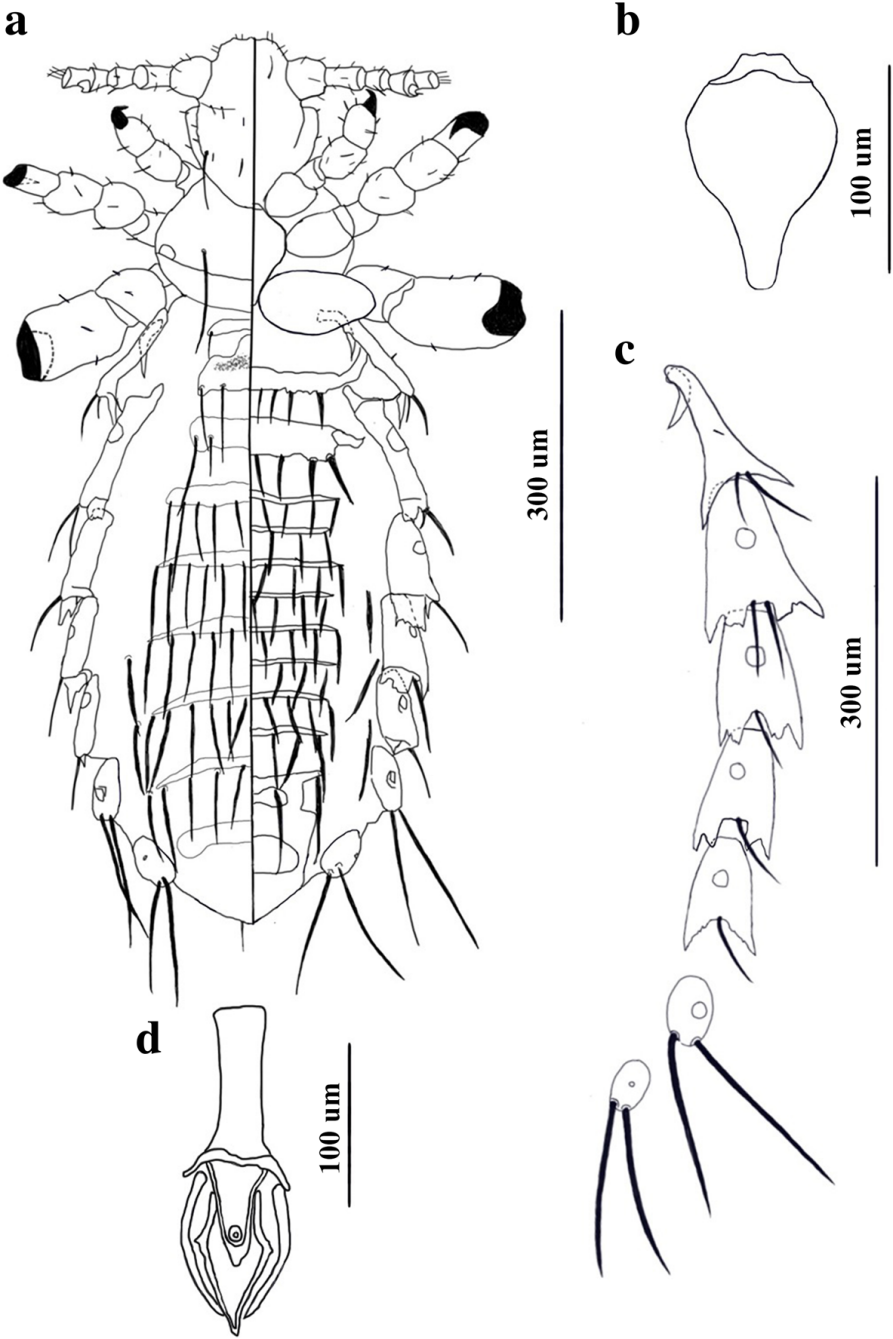
*Hoplopleura villosissima* n. sp. Male. **a** Habitus (dorsal/ventral view). **b** Thoracic sternal plate. **c** Paratergal plates. **d** Genitalia

*Male* [Based on 9 specimens; Fig. 1a.] Body length 862–1066 (942). Head longer than wide. Pre-antennal region short. Distal seta on dorsal surface of antennal segment 3 not sexually dimorphic. Apical head setae (ApHS) 4, anterior marginal head setae (AnMHS) 4. Dorsally, 4 sutural head setae (SuHS). Dorsal marginal head setae (DMHS) 4 on each side, second and third shifted medially. Small dorsal accessory head setae (DAcHS) 2, small dorsal anterior central head setae (DAnCHS) 2, small dorsal posterior central head setae (DPoCHS) 2, large dorsal principal head setae (DPHS) 2. Ventrally, 2 ventral principal head setae (VPHS).

Thorax wider than long, with 1 dorsal principal thoracic seta (DPTS) on each side, DPTS length 87.9–97.7 (88.9). Thoracic sternal plate (Fig. 1b) shield-shaped with squarish anterior process and elongate posterior process. Forelegs small, with small acuminate claws; midlegs and hindlegs progressively larger with correspondingly more robust tibio-tarsal claws.

Abdomen wider than thorax. Dorsally, 1 tergite per segment except for segment 3 with 2 tergites. Tergite 1 with 1 pair of small tergal abdominal setae (TeAS) posterolaterally. Tergite 2 with 2 pairs of TeAS posterolaterally, lateral one slightly shorter. Tergite 3 with 2 pairs of TeAS, with seta much longer on each side. Tergite 4 with 5 pairs of TeAS. Tergites 5–7 each with 5–6 pairs of TeAS. Tergte 8 with 4 pairs of TeAS. Tergites 6–8 each with 1 pair of dorsal lateral abdominal setae (DLAS). Tergite 9 without setae. Ventrally, no sternite on segment 1. Segment 2 with 1 sternite elongated laterally to articulate with paratergal plate and with 4 pairs sternal abdominal setae (StAS), lateral one stouter on each side. Segment 3 with 2 sternites, anterior one much larger, partially articulating with paratergal plate and with 7 StAS, the lateral 2 pairs larger and stouter than others. Sternites 4–10 narrow, each with 3–4 pairs of StAS. Sternites 6, 8 and 10 each with 1 pair of associated ventral lateral abdominal setae (VLAS). Paratergal plates (Fig. 1c) present on abdominal segments 1–8. All plates differentially sclerotized. Paratergal plate I small and offset medially. Paratergal plates II-VI each with 2 posterior lobes. Paratergal plate II with 1 small medial seta, 2 large posterior setae and acuminate posterior lobes. Paratergal plate III with 2 large setae and serrated posterior lobes. Paratergal plates IV, V and VI each with 1 large seta ventrally. Paratergal plates IV and V each with serrated posterior lobes; paratergal plate VI with acuminate posterior lobes. Spiracle diameter of segment 5 17.6–19.5 (18.4). Paratergal plates VII and VIII each with 2 long setae and lacking pointed posterior lobe.

Genitalia (Fig. 1d). Subgenital plate (Fig. 1a) with narrow anterolateral extension on each side and two lacunae; posterior lacuna larger than anterior lacuna. Basal apodeme slightly longer than parameres. Parameres uniformly sclerotized, with pseudopenis tapering to a point extending beyond apices of parameres (Fig. 1d).

**Fig. 2:**
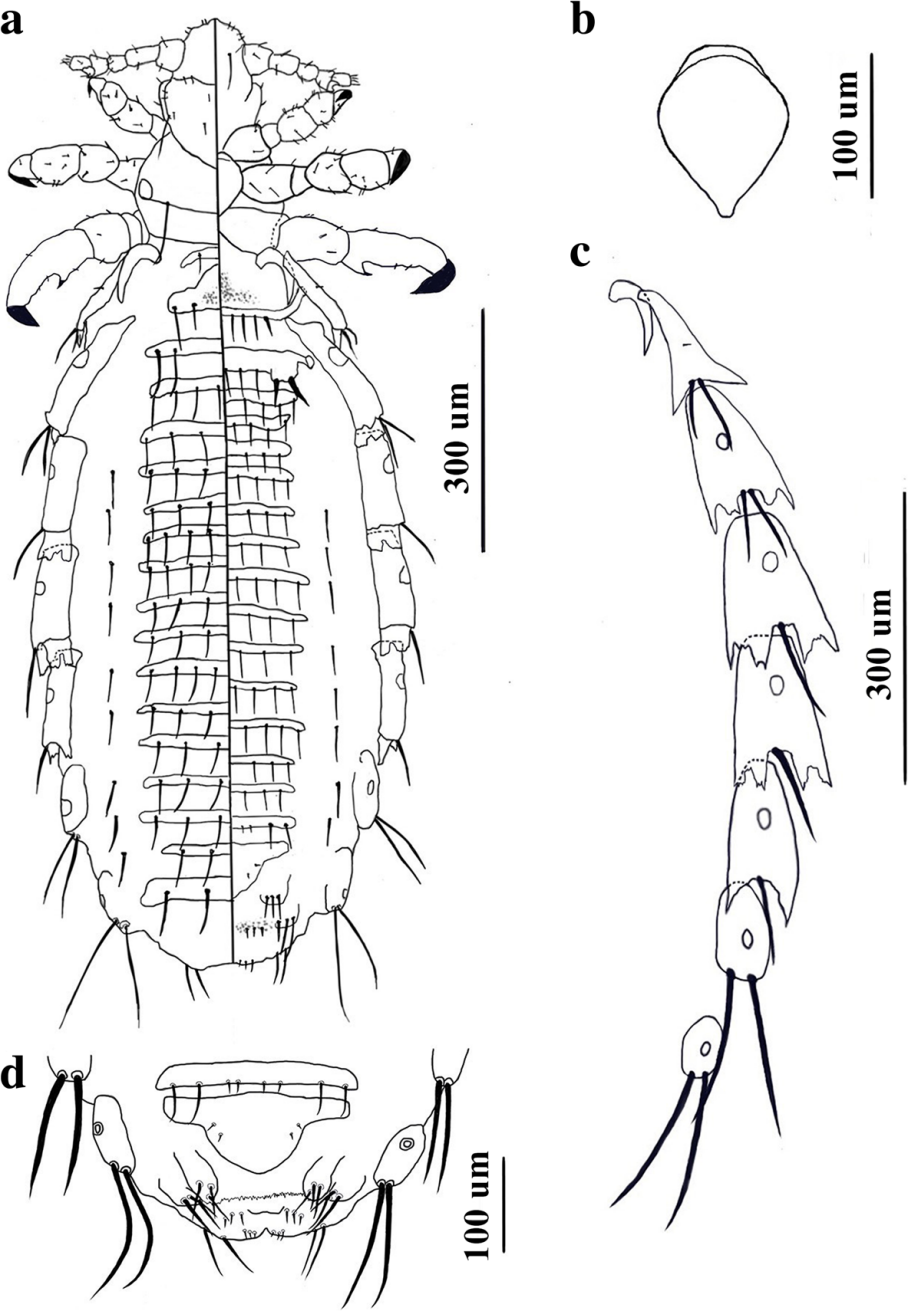
*Hoplopleura villosissima* n. sp. Female. **a** Habitus (dorsal/ventral view). **b** Thoracic sternal plate. **c** Paratergal plates. **d** Genitalia

*Female* [Based on 12 specimens; Fig. 2a.] Body length 1196–1348 (1225). Head longer than wide. Pre-antennal region short. ApHS 4, AnMHS 4. Dorsally, 4 SuHS. 4 DMHS on each side, the second and third of which are shifted medially. 2 small DAcHS, 2 small DAnCHS, 2 small DPoCHS and 2 large DPHS. Ventrally, 2 VPHS.

Thorax wider than long, with 1 DPTS per side. DPTS length 87–106.5 (96.7). Thoracic sternal plate (Fig. 2b) shield-shaped with broadly rounded anterior margin and elongate but small blunt posterior process. Legs approximately as in male.

Abdomen wider than thorax. Dorsally, 3 tergites per segment except for segments 1, 2 and 8, which have 1 tergite. Tergite of segment 1 with 1 pair of small TeAS posterolaterally. Tergite of segment 2 with 2 pairs of TeAS posterolaterally, lateral pair of TeAS slightly shorter. Anterior tergite of segment 3 with 2 pairs of TeAS, medial sternite with 7 TeAS; posterior tergite with 3 pairs of TeAS. Anterior and medial of segment 4 with 3 pairs of TeAS and 1 pair of DLAS, posterior tergite with 3 pairs of TeAS. Segment 5 anterior tergite with 4 pairs of TeAS and 1 pair of DLAS; medial tergite with 3 pairs of TeAS and with 1 pair of DLAS; posterior tergite with 3 pairs of TeAS. Anterior tergite of segment 6 with 4 pairs of TeAS and 1 pair of DLAS; medial tergite with 3 pairs of TeAS and 1 pair of DLAS; posterior tergites with 5 TeAS. Anterior and medial tergites of segment 7 both with 5 TeAS and 1 pair of DLAS; posterior tergite with 2 pairs of TeAS and 1 pair of DLAS. Segment 8 tergite with 2 pairs of TeAS. Ventrally, no sternite on segment 1. Segment 2 with 1 sternite articulating with paratergal plate II on each side and with 4 pairs of StAS, lateral ones stouter than others. Segments 3–7 each with 3 sternites. Anterior sternite of segment 3 with 7 StAS, lateral 2 pairs larger and stouter than others, inserted on sclerotized projection of posterolateral edge of sternite. Medial sternite with 7 StAS, posterior sternite of segment 3 with 4 pairs of StAS. Anterior and medial sternites of segment 4 both with 7 StAS, posterior sternite of segment 4 with 4 pairs of StAS and 1 pair of VLAS lateral to edge of posterior sternite. Segment 5 with 3 sternites, anterior one with 7 StAS, and 1 pair of VLAS; medial sternite with 4 pairs of StAS and 1 pair of VLAS; posterior sternite with 7 StAS. Anterior sternite of segment 6 with 7 StAS and 1 pair of VLAS, medial and posterior sternites both with 4 pairs of StAS, and with 1 pair of VLAS. Segment 7 with 3 sternites, anterior and medial sternites both with 7 StAS, posterior sternite with 4 pairs of StAS, which vary in size. Both medial and posterior sternites with 1 pair of associated VLAS. Paratergal plates (Fig. 2c) present on abdominal segments 1–8, all paratergal plates differentially sclerotized. Paratergal plate I small and offset medially. Paratergal plates II-VI all with 2 posterior lobes. Paratergal plate II with 2 large setae, 1 small seta medially and with acuminate posterior lobes. Paratergal plate III with 2 large setae and serrated posterior lobes. Paratergal plates IV-VI each with one large seta ventrally. Two specimens with minute setae on dorsal surface of paratergal plates IV-VI. Paratergal plates IV and V both with serrated posterior lobes. Spiracle diameter of segment 5 18.6–21.5 (20). Paratergal plate VI with acuminate posterior lobes. Paratergal plates VII and VIII each with 2 large long setae and lacking posterior lobe. Spiracles present on paratergal plates II–VIII, very small spiracle on paratergal plate VIII.

Genitalia (Fig. 2d) with subtriangular subgenital plate with 2 small mediolateral setae on each side. Gonopods VIII and IX distinct; gonopods VIII with 3 posterior setae subequal in size; gonopods IX with 3 posterior setae of differing lengths, lateral seta longest and medial seta shortest. Vulvar fimbriae indistinct. Three small setae on each side medial to gonopods IX.

### Diagnosis

The third antennal segment of *H. villosissima* n. sp. is not sexually dimorphic, which is different from *Hoplopleura irritans* Kuhn & Ludwig, 1967 [8]. The dorsal marginal head setae (DMHS) of the new species are not aligned; this is different from *Hoplopleura gyomydis* Kuhn & Ludwig, 1967 [8] and *Hoplopleura mastacomydis* Kuhn & Ludwig, 1967 [8] in which the DMHS are aligned in a row. Paratergal plate II of the new species has a small central seta and two large posterior setae, which differentiates it from *Hoplopleura pacifica* Ewing, 1924, *Hoplopleura bidentata* Neumann, 1909, *Hoplopleura cornata* Kim, 1972 [9], *Hoplopleura zyzomydis* Weaver, 2008 [10] and *Hoplopleura notomydis* Weaver, 2017 [11], which all lack the small central seta on paratergal plate II. *Hoplopleura villosissima* n. sp. has only one posterior seta on parategal plates IV-VI, whereas *H. gyomydis*, *H. mastacomydis*, *Hoplopleura uromydis* Kuhn & Ludwig, 1967 [8], *Hoplopleura calabyi* Johnson, 1960 [7], *H. notomydis* and *Hoplopleura melomydis* Weaver, 2017 [11], all have two setae on the posterior margin of paratergal plates IV-VI. The new species lacks posterior lobes on paratergal plate VII, which distinguishes it from *H. pacifica*, *H. mastacomydis*, *H. calabyi*, *H. zyzomydis*, *H. melomydis* and *Hoplopleura setosa* Weaver, 2017 [11]. The spiracles on the paratergal plates of the new sepcies are medium in size, smaller than in *H. uromydis* and *H. bidentata*, but larger than in *H. gyomydis*. *Hoplopleura villosissima* n. sp. can be further differentiated from *H*. *uromydis* by the serrated posterior lobes on its paratergal plates III-V. The posterior lobes on paratergal plates III-V of the new species are bilobate, which differs from the undivided lobes in *H. gyomydis*. *Hoplopleura villosissima* n. sp. has two setae on paratergal plate III, whereas *H. mastacomydis* has one short, stout seta inserted posteriorly. The sternal plate of the new species is shield-shaped whereas in *H. calabyi* it is elongated both anteriorly and posteriorly. The female of *H. villosissima* n. sp. has three sternites on abdominal segments 4–6 whereas the female of *H. bidentata* has two sternites on these segments. The female of *H. villosissima* n. sp. has three sternites on abdominal segment 7, in contrast to a single narrow sternite in the female of *H. zyzomydis* on this segment.

### *Polyplax spinulosa* collections from *R. villosissimus* and *R. rattus*

#### *Voucher material examined:*

Specimens ex *Rattus villosissimus*: 2♂ and 2♀, ex QM JM4825, Sandringham, Queensland, Australia (23°56'S, 138°47'E), 29.vii.1984, unknown collector; 1♂ and 2♀, ex QM JM4824, same location as for the holotype of *H. villosissima*; 2♂ and 3♀, ex QM JM 10742, Diamantina Lakes, Queensland, Australia (23°40'S, 141°5'E), 10.viii.1981, unknown collector; and 1♂, ex QM JM 5234, Marked Tree Waterhole, Queensland, Australia (23°17'S 138°9'E), 8.viii.1985. Specimens ex *Rattus rattus*: 3♂ and 4♀, ex Melbourne Museum Z65055, Grampians National Park, Victoria, Australia, November 2017, collector: Kevin Rowe.

### Remarks

*Polyplax spinulosa* Burmeister, 1839 is very similar in morphology to *Polyplax serrata* Burmeister, 1839 and *Polyplax reclinata* Nitzsch, 1864, but is distinct from other *Polyplax* species [17]. We rely on the following characters to identify *P. spinulosa* from *R. villosissimus* and *R. rattus*. *Polyplax spinulosa* is larger than *P. serrata* in body size and also has a shield-shaped sternal plate with a broadly rounded anterior margin. The sternal plate has a rounded anterior margin and an elongated posterior extension in *P. serrata* but a flat anterior margin and an elongated posterior extension in *P. reclinata*. The spiracles of the paratergal plates of *P. spinulosa* are smaller than those of *P. reclinata*. The setae on the third paratergal plate of *P. spinulosa* are subequal in size, which differentiates this species from *P. serrata* which has longer setae on the ventral surface [17]. The setae on each paratergal plate from III-VI are shorter than the paratergal plate itself in *P. spinulosa*, whereas in *P. serrata*, the setae on paratergal plate IV are as long as, or longer than paratergal plate IV [18, 19].

## Discussion

This article is the first to document sucking lice from the Australian long-haired rat, *R. villosissimus*. A new species of sucking louse, *H. villosissima*, is described and recorded. The new species can be identified by a combination of the following morphological characters: (i) the distal seta on the dorsal surface of antennal segment 3 is not sexually dimorphic; (ii) the dorsal marginal head setae are not in a row; (iii) the shield-shaped thoracic sternal plate; (iv) an additional small seta is present on paratergal plate II; (v) each of paratergal plates IV-VI has a large ventral seta; (vi) each of paratergal plates III-V has 2 serrated posterior lobes; and (vii) paratergal plate VI has 2 acuminate posterior lobes.

The description of *H. villosissima* n. sp. increases the total number of *Hoplopleura* species known in Australia from 12 to 13; this number includes an introduced species, *H. pacifica*, on the introduced black rat, *R. rattus* [12, 20]. The 12 *Hoplopleura* species from Australian native rodents are highly host specific. Only *H. irritans* has been found on two host species, *Rattus fuscipes* Waterhouse and *Rattus lutreolus* Gray [8]. The other 11 species, including *H. villosissima* n. sp., are found on one rodent species each. Most Australian native rodents are known to be parasitized by one sucking louse except for *Notomys alexis* Thomas which is parasitized by two *Hoplopleura* species, *H. notomydis* and *H*. *setosa* [11].

*Rattus villosissimus* hosts two species of sucking lice from different genera, *Hoplopleura* and *Polyplax*. This study is the first to record a *Polyplax* species from a native Australian rodent, and also the first record of two species of sucking lice from different genera and different families (Hoplopleuridae and Polyplacidae) from a species of Australian native rodent. Although Calaby & Murry [21] mentioned briefly that the spiny rat louse, *P. spinulosa*, was also found on some native *Rattus* species in settled areas in Australia, only the introduced brown rat and black rat, *Rattus norvegicus* Berkenhout and *R. rattus*, were recorded as the hosts of *P. spinulosa* in Australia [12, 17]. The only other record of a *Polyplax* species in Australia is for *P. serrata*, from the introduced house mouse, *Mus musculus* Linnaeus [12]. *Polyplax spinulosa* is less host-specific than most other sucking lice and has been found globally on eight species of rodents: *R. norvegicus* (type-host), *Bandicota bengalensis* Gray, *R. rattus*, *Rattus pyctoris* (Hodgson) (listed as *Rattus turkestanicus*), *Rattus nitidus* Hodgson, *Rattus argentiventer* Robinson & Kloss, *Rattus tanezumi* Temminck and *Rattus exulans* Peale, as reported by Durden & Musser [3].

**Fig. 3:**
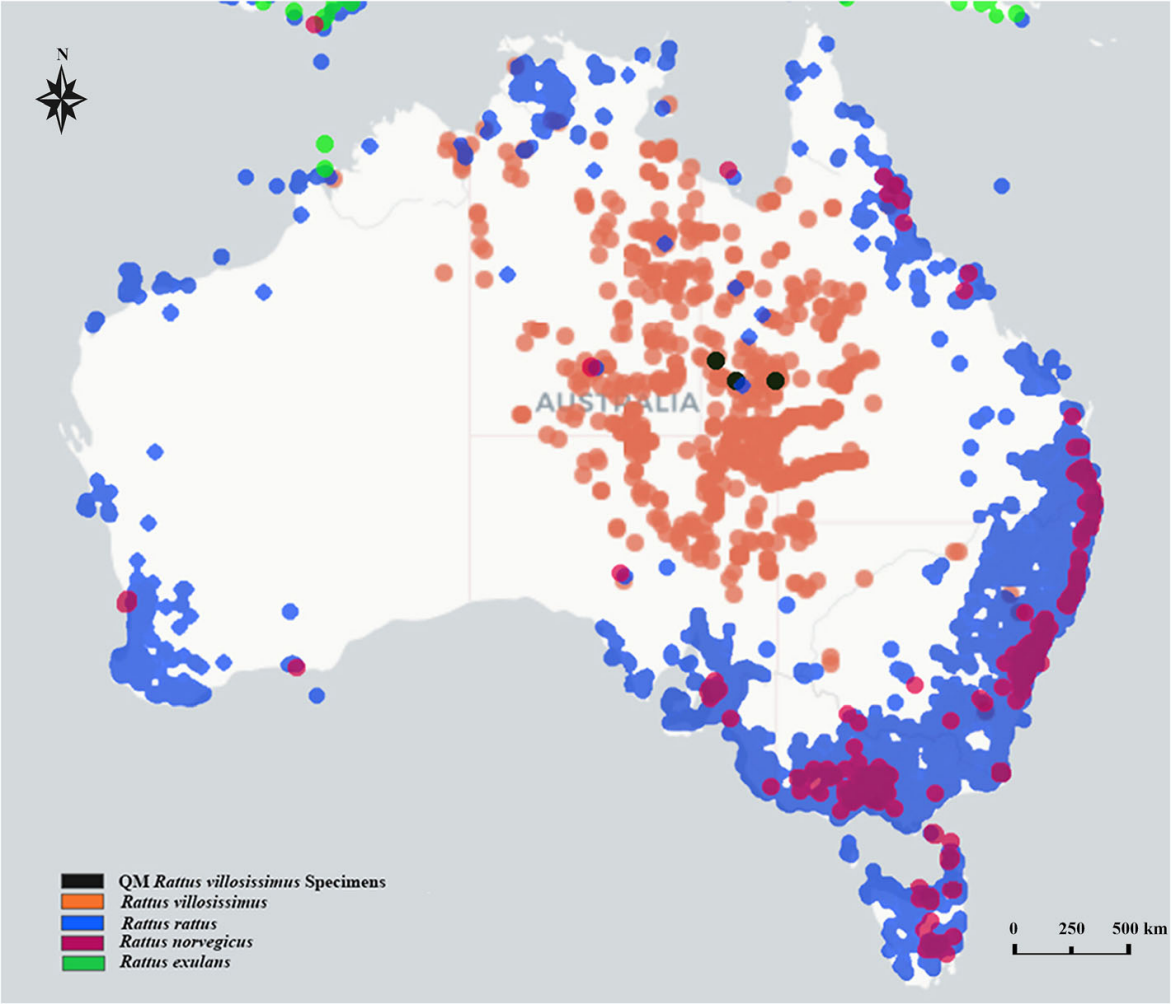
Distribution of *Rattus villosissimus*, *Rattus rattus*, *Rattus norvegicus* and *Rattus exulans* in Australia and the collection localities for Queensland Museum specimens (Adapted from https://www.ala.org.au)

*Rattus rattus*, known as the black rat, ship rat, or roof rat, is widespread around the world. As a reservoir host, *R. rattus* spreads parasites, pathogenic bacteria, protozoa and viruses, some of which are vector-borne, that adversely affect humans and wildlife [22]. *Rattus rattus* arrived in Australia by ships from Europe from the 1600s [22] and is widely distributed along coastal areas and in some inland areas where it overlaps with *R. villosissimus* in distribution (Fig. 3). Furthermore, both *R. rattus* and *R. villosissimus* are nocturnal and overlap in their diets [23]. *Rattus villosissimus* is well known for its population eruptions in arid areas of Australia and, because of its abundance during these periods, it has been assigned a common name of “the plague rat” [23]. *Rattus villosissimus* has been recorded in New South Wales, Queensland, Northern Territory, Western Australia, and South Australia (Fig. 3). Specimens of *R. villosissimus* used in the present study were collected from an inland area of Queensland where *R. rattus* also occurs (Table 1 and Fig. 3). Of the four introduced species of Muridae in Australia (*M. musculus*, *R. exulans*, *R. norvegicus* and *R. rattus*), only *R. norvegicus* and *R. rattus* were known to host *P. spinulosa* [12]. The brown rat, *R. norvegicus*, is distributed in coastal urban areas and rarely overlaps with *R. villosissimus* (Fig. 3). As sucking lice cannot survive for more than a few hours off the host, transfer of sucking lice is usually *via* physical contact between hosts [24]. It is very likely that transfer of *P. spinulosa* occurred from *R. rattus* to *R. villosissimus* in the inland areas where these two rodent species have the opportunities to contact physically. It would be interesting to investigate if *P. spinulosa* has transferred from *R. rattus* to other *Rattus* species that are endemic to Australia, and furthermore why *P. spinulosa* is more capable to parasitise different hosts than other sucking lice.

**Table 1:** Specimens of *Rattus villosissimus* in Queensland Museum

Registration number	Locality	Collection coordinates	Collection date	Sex
JM4810	Sandringham (61-22), Montara Dune	23°56'S, 138°47'E	28-Jul-84	Male
JM4823	Sandringham (61-22), Montara Dune	23°56'S, 138°47'E	29-Jul-84	Female
JM4824	Sandringham (61-22), Montara Dune	23°56'S, 138°47'E	29-Jul-84	Female
JM4825	Sandringham (61-22), Montara Dune	23°56'S, 138°47'E	29-Jul-84	Female
JM4832	Sandringham (61-22), Montara Dune	23°56'S, 138°47'E	29-Jul-84	Female
JM5234	Marked Tree Waterhole, 2 km North	23°17'S, 138°9'E	8-Aug-85	Unknown
JM10742	Diamantina Lakes	23°40'S, 141°5'E	10-14 Aug-81	Unknown

## Conclusions

A new species of sucking louse, *Hoplopleura villosissima*, and a new host record of the almost cosmopolitan spiny rat louse, *Polyplax spinulosa* from *R. villosissimus* are described in the present study. These are the first record of sucking lice from *R. villosissimus* and the first record of *Polyplax* species from a native Australian rodent. Because *R. villosissimus* overlaps with *R. rattus* in distribution but not with *R. norvegicus*, we propose that *P. spinulosa* transferred to *R. villosissimus* from *R. rattus*.

## Abbreviations

AnMHS: Anterior marginal head setae
ApHS: Apical head setae
DAcHS: Dorsal accessory head setae
DAnCHS: Dorsal anterior central head setae
DLAS: Dorsal lateral abdominal setae
DMHS: Dorsal marginal head setae
DPHS: Dorsal principal head setae
DPoCHS: Dorsal posterior central head setae
DPTS: Dorsal principal thoracic setae
StAS: Sternal abdominal setae
SuHS: Sutural head setae
TeAS: Tergal abdominal setae
VLAS: Ventral lateral abdominal setae
VPHS: Ventral principal head setae
